# Application of the novel tracer mitoxantrone hydrochloride in lymph node visualization during lateral neck dissection for thyroid carcinoma

**DOI:** 10.3389/fendo.2025.1712810

**Published:** 2025-12-12

**Authors:** Wu Li, Hui Li, Yulong Tang, Peng Wu, Shiwei Zhou, Xiaohua Song, Zeyang Liu, Zhiyuan Wang, Feng Shi, Xibin Xia, Zhaodong Ai, Xiaowei Peng

**Affiliations:** 1 Department of Thyroid Surgery, Hunan Cancer Hospital & The Affiliated Cancer Hospital of Xiangya School of Medicine, Central South University, Changsha, China; 2 Department of Ultrasound Diagnosis, Hunan Cancer Hospital & The Affiliated Cancer Hospital of Xiangya School of Medicine, Central South University, Changsha, China; 3 Department of Thyroid Internal Medicine, Hunan Cancer Hospital & The Affiliated Cancer Hospital of Xiangya School of Medicine, Central South University, Changsha, China; 4 Department of Diagnostic Radiology, Hunan Cancer Hospital & The Affiliated Cancer Hospital of Xiangya School of Medicine, Central South University, Changsha, China

**Keywords:** papillary thyroid carcinoma, neck dissection, mitoxantrone hydrochloride, lymph node tracer, staining efficacy

## Abstract

**Background:**

In this study, a novel lymph node tracer—mitoxantrone hydrochloride injection for tracing (MHI)—was intraoperatively administered into suspected metastatic lateral cervical lymph nodes of patients with papillary thyroid carcinoma (PTC), aiming to evaluate its staining efficacy and clinical value in enhancing the completeness of lateral neck dissection (LND).

**Methods:**

A prospective controlled study was conducted at Hunan Cancer Hospital between December 2023 and May 2024, enrolling a total of 67 patients diagnosed with thyroid carcinoma. After informed consent, patients were allocated to either MHI or methylene blue group. Subjects were divided into an MHI group (*n* = 42) and a methylene blue group (*n* = 25). All patients underwent LND.

**Results:**

The results demonstrated no statistically significant differences between the two groups in terms of operative time, total number of lymph nodes dissected, cervical drainage volume, or postoperative length of hospital stay (*P* > 0.05). However, the MHI group exhibited superior staining of suspected metastatic lymph nodes, with a greater number of metastatic lymph nodes and a higher positive ratio compared to the methylene blue group (*P* < 0.05).

**Conclusion:**

MHI causes minimal tissue contamination and can effectively enhance the thoroughness of LND in patients with thyroid carcinoma, demonstrating high clinical application value.

## Introduction

Thyroid carcinoma is the most common malignancy of the endocrine system, with papillary thyroid carcinoma (PTC) representing the predominant histological subtype, accounting for approximately 85%–98.5% of all thyroid cancers. Although PTC is relatively indolent in nature, it tends to metastasize to regional lymph nodes at an early stage (1). According to the National Comprehensive Cancer Network (NCCN) guidelines, the incidence of cervical lymph node metastasis at initial diagnosis in PTC patients ranges from 20% to 30%, with 28%–33% of these metastases being occult and difficult to detect intraoperatively, leading to missed positive lymph nodes (2). Therefore, complete dissection of cervical lymph nodes is particularly critical in the surgical management of thyroid carcinoma. Improving the detection accuracy of lateral neck lymph nodes through optimized staging, individualized treatment, and reduced residual disease can ultimately improve the recurrence-free survival (RFS) and quality of life for patients with thyroid cancer (3, 4).

In 2021, mitoxantrone hydrochloride (MHI), a novel bio-self-assembling nanocrystalline lymph node tracer, was approved for the first time by the National Medical Products Administration (NMPA) for use in lymph node visualization during thyroid cancer surgery. This provided a new clinical solution for lymph node tracing and improving the dissection rate of metastatic lymph nodes (5). However, as an emerging lymph node tracer, there is currently a lack of clinical studies evaluating its application in LND. Therefore, the present study aims to investigate the clinical value and advantages of MHI in lateral neck dissection (LND) for thyroid carcinoma, with the goal of providing robust evidence-based support for its clinical implementation.

## Subjects and methods

### Study subjects

A total of 67 patients diagnosed with PTC and scheduled to undergo LND between December 2023 and May 2024 at the Thyroid Surgery Department of Hunan Cancer Hospital/Affiliated Cancer Hospital of Xiangya School of Medicine, Central South University, were enrolled in this study. Among them, 20 were male and 47 were female. After informed consent, patients were allocated to either MHI or methylene blue group according to a pre-generated 2:1 block-randomisation list; the final allocation respected patient preference to minimise drop-out (pseudo-randomised design). The patients were divided into two groups: the MHI group (*n* = 42) and the methylene blue group (*n* = 25). All patients and their families signed written informed consent forms for the surgical procedures after being fully informed of the study details. This study was approved by the Medical Ethics Committee of Hunan Cancer Hospital (Approval No.: 2023105). The target sample size was calculated on the basis of a pilot study (n = 20) showing a mean difference of 6 positive nodes between groups. With α = 0.05 (two-sided), power = 0.80, and a 2:1 allocation ratio.

### Surgical protocol

All patients underwent LND. In the MHI group, 0.15 ml of MHI was injected under ultrasound guidance into lymph nodes in the lateral neck suspected of metastasis (hereinafter referred to as target lymph nodes) with a short axis greater than 3 mm. The injection procedure was identical in the methylene blue group. The injection protocol was as follows: One hour before surgery, 0.15 ml of the corresponding reagent was drawn into a 1 ml syringe. Under real-time ultrasound guidance., the needle was inserted directly into the lymph node. Before injection, negative aspiration was performed to prevent inadvertent intravascular administration of the dye. After the injection, the syringe was slowly withdrawn with a negative pressure. Gentle pressure was applied to the injection site using sterile gauze for 5 minutes. Strict procedural adherence is critical to avoid extravasation. Following lymph node tracing in the lateral neck, LND for thyroid carcinoma was performed. We conducted compartment-oriented lymph node dissection for papillary thyroid carcinoma, strictly following anatomical landmarks including the jugular vein, carotid bifurcation, and vagus nerve. We therefore clarify that our technique is “image-validated, tracer-enhanced LND”. In this study, all regional lymph node dissections were performed by the same surgical team led by a senior-level attending surgeon to minimize bias.

### Observation indicators

#### 1 Clinical indicators

General clinical parameters, including operative time, intraoperative blood loss, postoperative cervical drainage volume, and length of hospital stay, were meticulously recorded for both groups. Operative time was measured in minutes (min), starting from the initial skin incision to the completion of wound closure. Intraoperative blood loss was estimated based on the number of blood-soaked gauze pads used during the procedure. Cervical drainage volume was measured as the total fluid collected in the postoperative drainage bottle, expressed in milliliters (ml). The drainage tube was removed when the daily cervical drainage volume was ≤30 ml. Postoperative hospital stay was calculated in days (d), beginning from the day of surgery to the day of discharge.

#### 2. Evaluation of tracing efficacy

The effectiveness of lymph node tracing was assessed based on lymph node staining scores and the success rate of tracing. The lymph node staining score criteria were as follows: A score of 0 indicated no color change in the target lymph nodes of the lateral neck; A score of 1 indicated partial or marginal light staining (blue) of the target lymph nodes in the lateral neck; A score of 2 indicated deep blue staining of the target lymph nodes in the lateral neck.

#### 3. Dissection efficacy

Based on routine postoperative pathological examination, the total number of lymph nodes dissected and the number of lymph nodes with confirmed metastatic involvement were recorded and analyzed for both groups.

#### 4. Assessment of surgical field clarity

The clarity of the stained region surrounding the target lymph nodes was evaluated by two different doctors from the above senior attending surgeon team using the Fromme Surgical Field Quality Scale (6). The scores reflecting the clarity of the operative field around the intraoperatively stained target lymph nodes were analyzed for both groups to assess the quality of intraoperative staining.

#### 5. Perioperative-related complications

Perioperative complications were closely monitored and recorded in both groups, including intraoperative and postoperative massive hemorrhage, facial weakness or facial paralysis, Horner’s syndrome, and lymphatic leakage.

### Statistical methods

Data were analyzed using SPSS 27.0 statistical software. Measurement data conforming to a normal distribution are expressed as x ± s. For comparisons of indicator levels between groups, two-sample (independent-samples) t-test was employed. For intergroup comparisons of categorical data such as adverse reactions and efficacy evaluations, the χ² test was used. A *P*-value of <0.05 was considered statistically significant.

## Results

### Baseline data analysis

This study had no missing data; all cases were included in the analysis. There were no statistically significant differences between the two groups in terms of baseline characteristics, including age, height, weight, BMI, sex, maximum tumor diameter, number of localized lymph nodes, TNM staging, and surgical method. The clinical profiles of patients in both groups were well matched (**Table 1**).

**Table 1:** Clinical baseline information of the two groups of patients.

Clinical characteristics	MHI group	Methylene blue group	*T* value	*P* value	Significance
Age (years, x ± s)	37.41 ± 13.07	41.12 ± 12.85	-1.131	0.263	*P*>0.05
Height (m, x ± s)	1.64 ± 0.08	1.60 ± 0.09	1.889	0.063	*P*>0.05
Weight (kg, x ± s)	63.38 ± 12.80	63.36 ± 12.09	0.006	0.995	*P*>0.05
BMI(x ± s)	23.49 ± 3.56	24.50 ± 3.39	-1.143	0.257	*P*>0.05
Sex (n)	n=42	n=25			
Male	14	6	χ²=0.652	0.419	*P*>0.05
Female	28	19			
Max. Tumor Diameter (cm, x ± s)	13.80 ± 6.06	12.72 ± 5.39	0.735	0.465	*P*>0.05
Number of localized lymph nodes					
1	14	4	χ²=3.103	0.375	*P*>0.05
2	12	11			
3	8	6			
≥4	8	4			
TNM stage					
I	36	21	χ²=0.003	0.848	*P*>0.05
II/III	6	4			
Surgical approach					
Endoscopic Surgery	19	7	χ²=1.960	0.161	*P*>0.05
Conventional (Open) Surgery	23	18			

### Analysis of surgery-related indicators

Comparison of surgery-related indicators: There was no statistically significant difference in operative time between the MHI group (226.29 ± 102.17 mins) and the methylene blue group (212.92 ± 104.22 mins) (*t* = 0.514, *P* = 0.609, > 0.05). No statistically significant difference was observed in intraoperative blood loss between the MHI group and the methylene blue group (*t* = -1.185, *P* = 0.245, > 0.05). Similarly, no significant difference was found in cervical drainage volume between the two groups (*t* = 0.434, *P* = 0.666, > 0.05). Postoperative hospital stay did not differ significantly between the MHI group and the methylene blue group (*t* = 0.503, *P* = 0.616, > 0.05). However, regarding the total number of positive metastatic lymph nodes, the MHI group (15.76 ± 10.60 nodes) had a significantly higher number of dissected positive nodes compared to the methylene blue group (7.68 ± 5.27 nodes) (*t* = 2.456, *P* = 0.017, < 0.05), and the positive rate was also higher (*t* = 3.112, *P* = 0.003, < 0.05) (**Table 2**). *Post-hoc* power calculation based on the primary end-point (mean number of metastatic nodes): 82% power at α=0.05. After adjusting for age, sex, largest tumor diameter, extrathyroidal extension, and the number of suspicious lymph nodes on pre-operative ultrasound, the general linear model identified surgical approach (MHI vs. Methylene Blue) as the only significant predictor of the number of positive lymph nodes (β = 7.8, 95% CI: 2.1–13.4, p = 0.008). Patients in the MHI group had a mean of 7.8 additional positive nodes compared with those in the Methylene Blue group.

**Table 2:** Comparison of surgery-related indicators between the two groups.

Indicator	MHI group (x ± s)	Methylene blue group (x ± s)	*T* value	*P* value	Significance
Operative Time (min)	226.29 ± 102.17	212.92 ± 104.22	0.514	0.609	*P*>0.05
Intraoperative Blood Loss (mL)	106.43 ± 82.94	145.20 ± 150.58	-1.185	0.245	*P*>0.05
Cervical Lymph Node Drainage Volume (mL)	237.86 ± 194.72	221.40 ± 115.63	0.434	0.666	*P*>0.05
Postoperative Hospital Stay (d)	3.64 ± 0.97	3.52 ± 0.90	0.503	0.616	*P*>0.05
Total Number of Dissected Lymph Nodes (nodes)	41.76 ± 23.94	40.60 ± 22.94	1.234	0.222	*P*>0.05
Total Number of Positive Metastatic Lymph Nodes (nodes)	15.76 ± 10.60	7.68 ± 5.27	2.456	0.017	*P* < 0.05
Positive Rate	0.43 ± 0.25	0.21 ± 0.13	3.112	0.003	*P* < 0.05

### Evaluation of target lymph node staining

**Figure 1:**
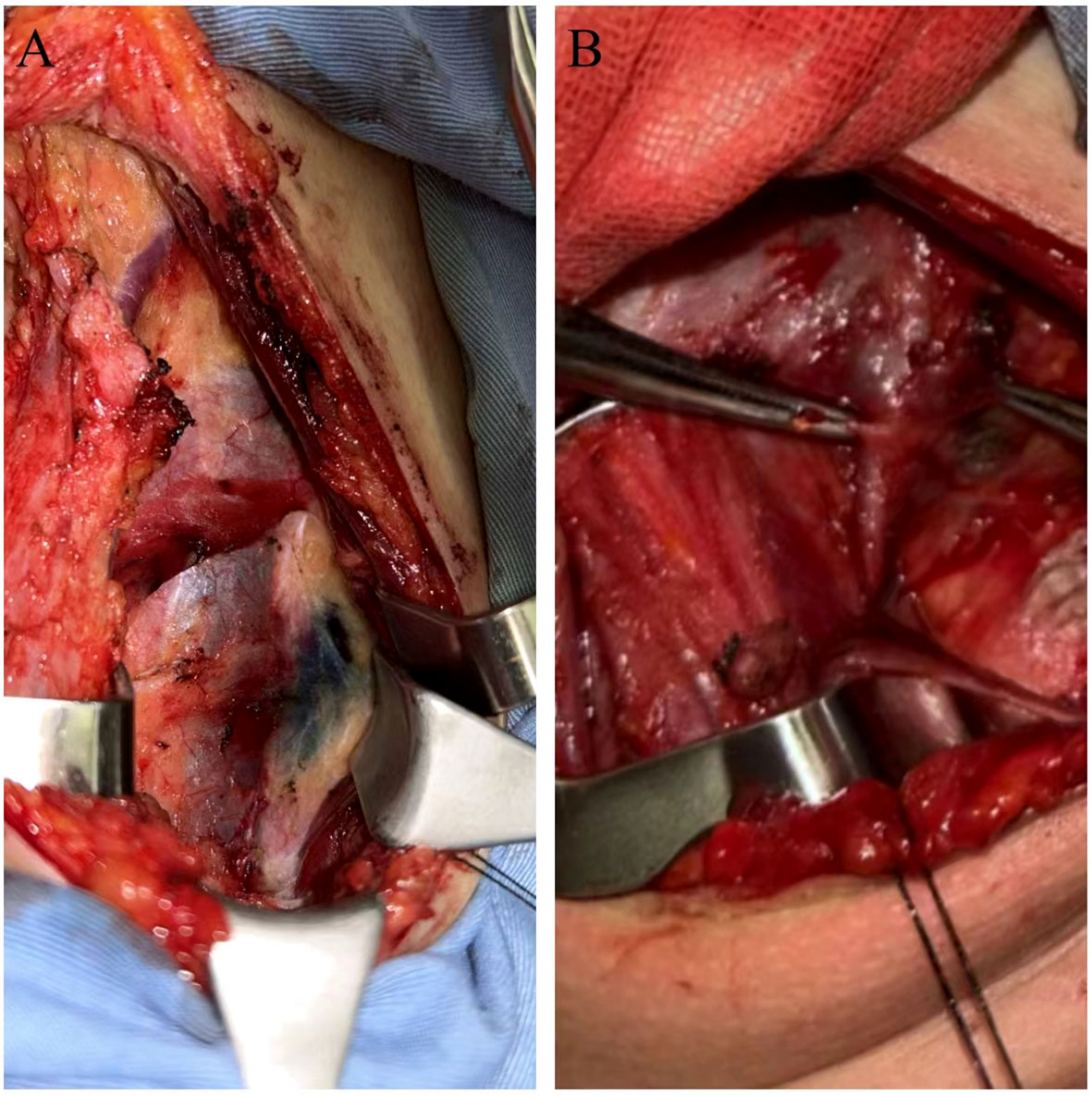
The operative field in the MHI and methylene blue group. The operative field of MHI group **(A)** and that of methylene blue group **(B)**.

There was no statistically significant difference between the MHI group and the methylene blue group in terms of target lymph node staining time. However, the operative field clarity score was significantly higher in the MHI group (**Figure 1A**) compared to the methylene blue group (**Figure 1B**), and the difference was statistically significant (*t* = 2.593, *P* = 0.015, < 0.05). Two surgeons blinded to group assignment scored staining independently (κ=0.82), indicating good reproducibility and reliability of the experimental results.

### Perioperative complications

There were no statistically significant differences between the two groups regarding the incidence of postoperative complications, including hoarseness, facial weakness or paralysis, Horner’s syndrome, and lymphatic leakage (all *P* values > 0.05).

## Discussion

Cervical lymph node metastasis is one of the key risk factors affecting the prognosis of PTC. At the time of diagnosis, 20%–90% of PTC patients already present with lymph node metastases, which may be difficult to detect intraoperatively, leading to potential omission of metastatic nodes. Multiple clinical studies have demonstrated that the completeness of lymph node dissection is closely related to the prognosis and recurrence rate of PTC (7). In this study, MHI, a novel bio-self-assembling nanocrystal tracer, was employed, while methylene blue served as a traditional blue biological dye. The results showed that the total number of positive metastatic lymph nodes dissected was higher in the MHI group (15.76 ± 10.60) compared with the methylene blue group (7.68 ± 5.27), with statistical significance (*t* = 2.456, *P* = 0.017, < 0.05). Further statistical analysis revealed a higher dissection rate of positive lymph nodes in the MHI group (*t* = 3.112, *P* = 0.003, < 0.05). The operation time of the MHI group was slightly longer (226 minutes vs 213 minutes), although there was no statistical difference, there was a trend. This may be related to the resection of more positive lymph nodes, as metastatic lymph nodes are usually more severely adherent to normal tissues. This study suggests that during LND for thyroid carcinoma, targeting lymph nodes using MHI can enhance the thoroughness of dissection, thereby helping to reduce the recurrence rate and improve prognosis.

Previous studies have shown that the primary component of MHI is originally an anthraquinone-based antineoplastic agent, which regulates DNA replication and has been used in the treatment of acute leukemia, lymphoma, prostate cancer, and breast cancer (8). After molecular structural modification, mitoxantrone hydrochloride undergoes a transformation under the altered pH conditions within the interstitial spaces of thyroid gland tissue, where drug molecules form spherical nanocrystals (approximately 100 nm in diameter) through non-covalent interactions. These nanocrystals are unable to traverse the capillary walls but can selectively enter lymphatic capillaries, accumulating in regional lymph nodes via lymphatic drainage. Once there, they are captured and retained by macrophages within the lymph nodes, thereby producing a blue-stained tracer effect. In contrast, the lymphatic tracing mechanism of methylene blue primarily relies on its nature as a blue dye that can be taken up by the lymphatic system and accumulate in lymph nodes, rendering them visibly blue. Following intraoperative injection of MHI into lateral cervical lymph nodes, a clear and distinct blue visualization can be rapidly achieved, forming a stark contrast with surrounding physiological tissues. The localization is precise, and the diffusion (smudging) effect is minimal. In cases of extravasation of MHI, the leaked dye can be effectively absorbed and removed using saline-soaked gauze. Conversely, leakage of methylene blue tends to cause extensive blue staining, which cannot be adequately eliminated even after irrigation with normal saline, thereby interfering with the surgical field due to diffuse staining. In this study’s evaluation of staining efficacy and contamination, the MHI group demonstrated a significantly superior tracer score and surgical field clarity score compared to the methylene blue group (*t* = 2.593, *P* = 0.015, < 0.05). It was also observed that the intraoperative injection dosage of methylene blue is difficult to control: an insufficient dose fails to achieve effective lymphatic tracing, whereas an excessive dose contaminates the operative field, obscuring the identification of surrounding normal anatomical structures and consequently increasing the risk of iatrogenic injury. There was no statistically significant difference in the incidence of perioperative complications between the two groups in this study, which can be attributed to the extensive experience of the operating surgeon (with an annual surgical volume exceeding 500 cases) (9–12). However, in surgeons with less experience or lower annual surgical volume, different outcomes may be observed. This represents an important scientific issue to be addressed in future research.

This study indicated that the number of metastatic lymph nodes identified in the MHI group was higher than that in the methylene blue group. Intraoperatively, the chromatic properties of MHI facilitated clearer visualization of lateral cervical lymph nodes, thereby enabling the surgeon to perform more regionally skeletonized dissections and to harvest a greater number of lymph nodes. Additionally, pathologists still rely on conventional methods to identify lymph nodes, which makes it difficult to distinguish and isolate lymph nodes smaller than 5 mm from surrounding adipose tissue by visual inspection alone (13). However, the superior staining efficacy of MHI can assist pathologists in identifying and isolating more lymph nodes, thereby demonstrating its advantages in terms of target specificity, visual clarity, and procedural convenience, and providing robust support for improving lymph node detection rates (14).

Perioperative observation revealed no severe complications or adverse drug reactions in either group, suggesting that despite MHI being composed primarily of an anti-tumor proliferation agent, its safety profile is comparable to that of methylene blue. In fact, Yang et al. reported that during sentinel lymph node tracing in breast cancer using MHI, no systemic adverse reactions were observed even when the injection volume was increased to 2.0 ml (15). The plasma concentration of mitoxantrone at that dosage ranged from 26 to 79.4 ng/ml, which corresponds to only 15% of the conventional dosage used for the treatment of acute leukemia, and no hepatotoxicity or nephrotoxicity was observed. Moreover, Chen Shaobo et al. reported in a phase I clinical trial that following intrathyroidal injection of MHI, the peak plasma concentration was reached within 10 minutes, and the drug was almost entirely eliminated from the plasma within 30 minutes. No serious adverse events occurred in the experimental group in the previous study, and the causes of adverse events were unrelated to the tracer, indicating that the tracer at a dose of 0.2-0.6 mL is relatively safe and generally does not cause systemic toxic side effects (5). The single injection dose in this study was only 0.15 ml, the total dose administered not exceeding 0.6 ml. This short systemic retention time helps to minimize drug accumulation in the body and reduces the potential risk of toxicity.

In summary, the findings of this study demonstrate that the application of MHI in LND for thyroid carcinoma offers greater thoroughness in lymph node clearance compared to methylene blue. Additionally, MHI provides superior chromatic enhancement, resulting in clearer surgical fields. Its clinical efficacy surpasses that of the methylene blue tracing protocol, offering evidence-based support for its broader clinical adoption. However, it must be admitted that patients were allocated by “informed preference”. This is an exploratory analysis without adjustment for multiple comparisons; positive findings require validation in a future large-scale randomized controlled trials to prove non-inferiority.

## Data Availability

The original contributions presented in the study are included in the article/supplementary material. Further inquiries can be directed to the corresponding authors.
